# Mobile app activity engagement by cancer patients and their caregivers informs remote monitoring

**DOI:** 10.1038/s41598-024-53373-w

**Published:** 2024-02-09

**Authors:** Reem Yunis, Stephanie J. Fonda, Sara Aghaee, Ai Kubo, Sharon W. Davis, Raymond Liu, Elad Neeman, Ingrid Oakley-Girvan

**Affiliations:** 1 Strategy and Science Departments, Medable Inc., 525 University Avenue, Suite A70, Palo Alto, CA 94301 USA; 2 Estenda Solutions, Inc., Wayne, PA USA; 3 Division of Research, Kaiser Permanente Northern California, Oakland, CA USA; 4 Department of Hematology Oncology, Kaiser Permanente Northern California, San Francisco, CA USA; 5 San Rafael Medical Center, Kaiser Permanente Northern California, San Rafael, CA USA

**Keywords:** Health care, Medical research

## Abstract

Mobile phone applications (“apps”) are potentially an effective, low-burden method to collect patient-reported outcomes outside the clinical setting. Using such apps consistently and in a timely way is critical for complete and accurate data capture, but no studies of concurrent reporting by cancer patient–caregiver dyads have been published in the peer-reviewed literature. This study assessed app engagement, defined as adherence, timing, and attrition with two smartphone applications, one for adult cancer patients and one for their informal caregivers. This was a single-arm, pilot study in which adult cancer patients undergoing IV chemotherapy or immunotherapy used the DigiBioMarC app, and their caregivers used the TOGETHERCare app, for approximately one month to report weekly on the patients’ symptoms and wellbeing. Using app timestamp metadata, we assessed user adherence, overall and by participant characteristics. Fifty patient–caregiver dyads completed the study. Within the one-month study period, both adult cancer patients and their informal caregivers were highly adherent, with app activity completion at 86% for cancer patients and 84% for caregivers. Caregivers completed 86% of symptom reports, while cancer patients completed 89% of symptom reports. Cancer patients and their caregivers completed most activities within 48 h of availability on the app. These results suggest that the DigiBioMarC and TOGETHERCare apps can be used to collect patient- and caregiver-reported outcomes data during intensive treatment. From our research, we conclude that metadata from mobile apps can be used to inform clinical teams about study participants' engagement and wellbeing outside the clinical setting.

## Introduction

There is increasing awareness that remote health monitoring can be beneficial during a clinical trial^1–3^ and for routine health care^4–6^. Smartphone applications (“apps”) that help patients track symptoms and general health through electronic patient-reported outcomes (ePROs) are potentially an effective and low-burden method to collect trial and healthcare data outside the clinical setting^7–12^. Longitudinal data capture via a smartphone app may be a powerful approach to monitor patient progress and/or adverse events in clinical trials^1,2^ or usual care^13,14^ without the need for frequent in-person visits to a clinic.

In this relatively new area, little has been documented regarding what works best to encourage participants to consistently use mobile app interventions^15–17^. High-quality data depends on the user to regularly enter accurate information; incomplete questionnaires can compromise the quality of ePRO data^18,19^.

The term “engagement” has been used to discuss the extent of mobile app utilization by users^20^. Although engagement has not been consistently measured^15^, it is primarily conceptualized with three components: behavioral, cognitive, and affective^21^. The behavioral component—the focus of this article—is generally measured quantitatively through physical interaction of the user with the mobile health system, including metadata captured by the app, adherence with app activities, number of log-ins, time spent on each activity, and number of features accessed and screens viewed^22,23^. Semi-structured interviews were completed in previously published usability studies of each app; these interviews addressed some of the cognitive and affective components of engagement^24,25^.

This project developed and tested DigiBioMarC^24^ a smartphone app for individuals with cancer (“patients”), and TOGETHERCare^25^, a smartphone app for informal caregivers, to collect health, activity, and psychosocial data about patients and caregivers frequently and easily.

Cancer treatment may result in adverse events that require dose reductions or other interventions, making frequent and timely reporting of patient symptoms important^26^. There is a large body of literature about the use of mobile health apps by patients to report their health status^27,28^, but to date no other studies of concurrent reporting by cancer patient–caregiver dyads have been published in the peer-reviewed literature.

The aim of this article is to explore behavioral engagement with apps that collect ePROs by reporting on the adherence of both patients and caregivers, focusing on whether cancer patients and caregivers completed app activities as planned, the amount of time spent on expected activities, and associations of participants’ characteristics to explore app burden and potential obstacles to engagement.

## Methods

### Study design and sample

This is a single-arm, prospective study in which adult cancer patients within the Kaiser Permanente Northern California (KPNC) system and their informal caregivers were asked to use the DigiBioMarC (for cancer patients) or TOGETHERCare (for caregivers) smartphone apps for 28 days after enrollment (the study period). KPNC is an integrated healthcare delivery system serving over 4.5 million members representative of the general Northern California population^29^. The KPNC Institutional Review Board reviewed and approved the study protocol.

Cancer patients scheduled to receive intravenous chemotherapy or immunotherapy during the study period, were identified using the KPNC electronic health record system and were recruited by email invitations from October 2020 to March 2021. Recruitment emails were sent to 2155 potential patients. Of the 247 respondents (11% of recruitment emails), 166 (67% of respondents) were determined to be ineligible (no iPhone (n = 33), no eligible/willing caregiver (n = 24), no scheduled IV therapy during the study (n = 13), not English speaking (n = 5), physician indicated a contradiction to participation (n = 42), invalid emails (n = 41), deceased (n = 2), ineligible unspecified (n = 6)), 20 declined to participate before eligibility could be confirmed, and 7 declined to participate after learning more details about the study. No background information was tabulated for those that did not consent to participate in the study as we were restricted under our IRB approved protocol to information from the EHR only for those individuals that provided informed consent and HIPAA authorization.

Recruitment and all methods were performed in accordance with the relevant guidelines and consent were completed remotely through a video or phone call. During this call, the protocol coordinator assisted interested and eligible participants in downloading the app and instructed them on how to review and sign the informed consent document in the app. No other training was provided in how to navigate the applications. Participants were required to provide written informed consent within the application prior to proceeding with the study. Both patients and caregivers who continued responding to activities in the app through the study period and completed the two semi-structured virtual interviews received a $100 gift certificate for their time and effort. Based on the IRB recommendations, data collected through the app were not relayed to the participating patients’ or caregivers’ clinical team. The exception was that the research associate was to be automatically alerted to survey scores for mental health measures from specific surveys if they exceeded a predetermined threshold. If an alert was received, the research associate was to call the participant, ask if they were in immediate danger and if so call 911 to request the police conduct a “health and welfare check.” If not in immediate danger but they expressed distress, the research assistant was to ask if they wanted a Kaiser clinician to talk with them.

Eligibility requirements for cancer patients included being 18 years of age or older, receiving intravenous chemotherapy or immunotherapy treatments at a KPNC medical facility at the time of the study, having an iPhone 6 or higher, and the ability to read and communicate in English^24^. Patients were also required to have an informal caregiver to be eligible to participate in the study. The informal caregivers were identified by the patient and screened for eligibility during recruitment. Caregivers did not need to be KPNC members but did need to be 18 years of age or older, have an iPhone 6 or higher, and able to read and communicate in English. There was no requirement for the length of time the caregiver had lived with the patient. Cancer patients with severe mental illness or insufficient cognition to consent (as determined by their physician) were not eligible.

### App activities and schedule

As previously described in greater detail^24,25^, the DigiBioMarC and TOGETHERCare apps were composed of “activities” defined as surveys as well as physical assessments. Surveys assessed the cancer patients’ symptoms and functioning, social and financial resources, wellbeing, and stress, among other measures from the perspective of the cancer patient (DigiBioMarC) and their caregiver (TOGETHERCare). Physical assessments, gait speed^30^ and “sit to stand”^31^ tasks, were to be completed by all patient participants but were not asked of caregivers.

Each time an app activity was made available to an individual participant, it counted as an “instance” of that activity. The number of instances varied for different activities. The study protocol expected, but did not mandate, participants to complete the activity within 24 h. Some activities were expected for completion only at the beginning of the study period (i.e., one instance), some at the beginning and end (i.e., two instances), and some were scheduled for completion either every 7 or 14 days (i.e., three to five instances). The intervals between instances of an activity were standardized such that after a user completed a planned activity, it could not be completed again before the minimum expected days had elapsed, depending on the planned interval. The activity remained open until it was completed. The frequency and timing of the app activities were intended to capture relevant health and wellbeing data pertaining to cancer patients. Research staff did not prompt participants by phone, email, or text to complete open activities. Instead, notifications or reminders were sent within the app to caregiver participants three times per week, and to patients every two to three days, midday on a preset schedule. These reminders were not linked to specific activities or whether the participant had completed them but were instead general reminders to complete any open activities.

While the active study duration was 28 days, for the purposes of this examination, we allowed activities to be completed within a 33-day window past enrollment to allow a few extra days for users to become familiar with the apps and study expectations.

### Measures

*Adherence.* Using the timestamp data (app operational metadata), the study assessed app adherence as: (1) completion of all expected app activities within 33 days; (2) completion of individual app activities; (3) completion of expected app activities within 24 and 48 h; and (4) average time to complete individual app activities at the beginning and end of the study.

Based on the expected instances, we calculated denominators for the percentages of app activities completed. The numerators for these percentages are the total completions (including all study participants within each group) over the entire active study period.

We measured the app time commitment as the minutes to complete each activity based on metadata, changes in completion time over the active study period, and total time at the beginning and end of the study period, when the greatest number of activities were expected.

*Potential obstacles to app adherence.* To provide contextuality of the participants’ adherence and potential app burden, we collected information from the patients’ KPNC medical records about the patients’ cancer status, namely, type, stage, and presentation of cancer at study enrollment. In the apps themselves, we collected information about the participants’ social demographics (age, gender, ethnicity, race, educational attainment, employment status) and the caregivers’ perceived amount of time that they provided care at the beginning and end of the study period. We conducted semi-structured interviews with participants on approximately the seventh day of app use and again after participants completed the final app surveys, using a secure videoconferencing program. The results of these surveys have been previously reported^24,25^.

### Analysis

The analysis is descriptive and exploratory. First, we summarized the baseline characteristics of the study participants. Second, we calculated summary statistics for the adherence measures and the average minutes spent on each activity. Third, we performed two-sided Fisher Exact tests to assess possible associations between the participant characteristics and completion of 80 + percent of the app activities within the active study period.

## Results

### Study participant baseline characteristics

Fifty of the 54 enrolled patient–caregiver dyads completed the study (3 patients stopped participating due to serious declines in health and one patient did not start the planned treatment making them ineligible). Seventy-six percent of the patients had advanced cancer (stage 3 or 4), and 28% had relapsed diagnosis as of their enrollment date. The caregivers in the enrolled dyads were predominantly male (62%) and spouses/partners of the cancer patients (78%), who were predominantly female (78%) (Table 1). Eighty percent of the patients were 50 years of age or older with about a quarter of them ≥ 70 years. The caregiver cohort was overall younger with 50% aged 50–69 years. Close to two-thirds of the participants (combined patients and caregivers) were white, and over 80% identified as non-Hispanic or Latino. Fifty-four percent of the caregivers and 70% of the patients had an associate degree or higher, respectively. Almost all caregivers (n = 48) and cancer patients (n = 49) had “adequate” cancer health literacy as indicated by the 6-Item Cancer Health Literacy Test (CHLT-6). Unemployment at the start of the study was higher in patients than in caregivers (68% vs 42%, respectively).

**Table 1:** Characteristics of study participants at baseline (N = 50 dyads).

Characteristic	Caregivers	Patients
	N = 50	N = 50
Age, mean years (SD)	54.4 (16.6)	59.9 (11.6)
Gender, female, n (%)	19 (38.0)	39 (78.0)
Ethnicity, n (%)		
Hispanic	4 (8.0)	4 (8.0)
Not Hispanic	41 (82.0)	43 (86.0)
Declined to answer	5 (10.0)	3 (6.0)
Race, n (%)		
White	32 (64.0)	34 (68.0)
Person of color	8 (10.0)	9 (18.0)
Multiracial/Other	7 (14.0)	6 (12.0)
Declined to answer	3 (6.0)	1 (2.0)
Marital status, n (%)		
Married/partnered	38 (76.0)	42 (84.0)
Single	8 (16.0)	1 (2.0)
Widowed, divorced or declined to answer	4 (8.0)	7 (14)
Education, n (%)		
≤ High school graduate/GED	5 (10.0)	4 (8.0)
Some college	18 (36.0)	11 (22.0)
Associate's or Bachelor’s degree	17 (34.0)	20 (40.0)
Graduate degree	10 (20.0)	15 (30.0)
Employment status, n (%)		
Employed full time	18 (36.0)	11 (22.0)
Self-employed/freelance or employed part time	6 (12.0)	5 (10.0)
Unemployed	7 (14.0)	5 (10.0)
Student, homemaker or on disability leave	5 (10.0)	5 (10.0)
Retired	14 (28.0)	21 (42.0)
Relationship to patient		
Spouse or partner	39 (78.0)	NA
Child or grandchild	6 (12.0)	NA
Parent or parent in-law or other relative or friend	5 (10.0)	NA
Cancer Health Literacy Test-6 (CHLT-6) score, mean (SD)	5.7 (0.5)	5.8 (0.5)
Cancer stage at diagnosis, n (%)		
1	NA	5 (10.0)
2	NA	7 (14.0)
3	NA	18 (36.0)
4	NA	20 (40.0)
Type of cancer, n (%)		
Breast	NA	17 (34.0)
Gastrointestinal	NA	12 (24.0)
Gynecologic	NA	12 (24.0)
Thoracic or skin	NA	7 (14.0)
Other	NA	2 (4.0)
Type of treatment, n (%)		
Chemotherapy	NA	34 (68.0)
Immunotherapy	NA	8 (16.0)
Multiple	NA	8 (16.0)

### Overall completion of expected app activities

The mean number of activities completed during the active study period was 24.2 out of 28, (standard deviation [SD] = 4.4) for patients (86% completion rate) and 35.6 out of 42 (SD = 7.1) for caregivers (84% completion rate) (Supplement 1). Seventy-six percent (76%) of patients and 66% of caregivers completed 80% or more of the expected activities, respectively. More activity completion in the caregiver group was related to more activity completion in the patient group (correlation coefficient = 0.5, *p* ≤ 0.001). No participant survey responses met the threshold to trigger a mental health email notification to the research assistant.

### Completion of individual app activities

One hundred percent of the “About You” surveys were completed by all study participants (Table 2). For the caregivers’ repeated surveys, the “Every Other Day” survey was least completed (77.1% of the expected surveys were completed) and the “Quick Check-in” (PHQ4) survey was most completed (93.0% were completed). For the cancer patients’ repeated activities, the “Gait Speed” assessment was the least completed (79.6% completion rate) and, again, the “Quick Check-in” survey was the most completed (95.0% completion rate). For both caregivers and patients, the completion rate of more frequent activities was lower compared with less frequent ones.

**Table 2:** Completion of TOGETHERCare and DigiBioMarC app activities during the active study period^a^.

Activity name in app (General content)	About	Total expected activities	Number (%) completed			Percent increase between 24 and 48 h
			Total	Within 24 h	Within 48 h	
Caregivers (n = 50) using TOGETHERCare						
About you (E = 1) (Demographics)	Self	50	50 (100.0)	46 (92.0)	47 (94.0)	2.2
Quick check-in (E = 2) (PHQ4)	Self	100	93 (93.0)	69 (69.0)	84 (84.0)	21.7
Care snapshot (E = 3) (Caregiving burden)	Self	150	131 (87.3)	92 (61.3)	111 (74.0)	20.7
Every other day (E = 14) (Sleep, exercise, fatigue, worry)	Self	700	540 (77.1)	–	–	–
Your wellness (E = 5) (PROMIS global health)	Self	250	217 (86.8)	152 (60.8)	176 (70.4)	15.8
Short symptom report (E = 5) (PRO-CTCAE)	Patient	250	214 (85.6)	150 (60.0)	170 (68.0)	13.3
Fast 4 (E = 5) (PROMIS physical function)	Patient	250	221 (88.4)	159 (63.6)	182 (72.8)	14.5
COVID-19 patient impact (E = 2) (COVID impact on patient care)	Patient	100	91 (91.0)	64 (64.0)	82 (82.0)	28.1
Cancer patients (n = 50) using DigiBioMarC						
About you (E = 1) (demographics)	Self	50	50 (100.0)	45 (90.0)	47 (94.0)	4.4
Quick check-in (E = 2) (PHQ4)	Self	100	95 (95.0)	73 (73.0)	85 (85.0)	16.4
Short symptom report (E = 5) (PRO-CTCAE)	Self	250	222 (88.8)	163 (65.2)	179 (71.6)	9.8
Fast 4 (E = 5) (PROMIS physical function)	Self	250	219 (87.6)	153 (61.2)	173 (69.2)	13.1
Gait speed (E = 5) (Gait and balance active assessment)	Self	250	199 (79.6)	130 (52.0)	150 (60.0)	15.4
Sit and stand (E = 5) (Sit to stand active assessment)	Self	250	202 (80.8)	130 (52.0)	153 (61.2)	17.7
COVID-19 your input (E = 2) (COVID-19 behavior, knowledge)	Self	100	94 (94.0)	71 (71.0)	86 (86.0)	21.1

*E* Expected number of times for an activity (assessment or survey), *PHQ4* Patient Health Questionnaire for anxiety and depression (4-item), *PROMIS* Patient-Reported Outcomes Measurement Information System, *PRO-CTCAE* Patient-Reported Outcomes-Common Terminology Criteria for Adverse Events with 12 selected symptoms chosen by the team’s oncologists (the 12 symptoms can be found in a prior publication^24^).

^a^“Total Expected Activities” were calculated by multiplying the number of times an activity was expected (E) by the number of participants. The “Within 24 h” and “Within 48 h” columns refer to the number and percentages of activities completed within 24 and 48 h, respectively, of when the activity was available to the participant through the app. The denominators for the percentages are the numbers in the “Total Expected Activities” column. The “Percent Change” calculations used the frequencies for the “Within 24 h” column as the base values.

Ninety percent or more of the “About You” surveys were completed within 24 h from the time it became available for both patients and caregivers (Table 2). Lower percentages of the weekly activities were completed within 24 h. For example, 60% of the “Short Symptom Reports” (PRO-CTCAE) surveys were completed by caregivers, and 52% of the “Gait Speed” and “Sit and Stand” physical assessments were completed by cancer patients within 24 h. Expanding the completion window to 48 h increased the percentage completed by a range of 2.2% (caregivers’ “About You”) to 28.1% (caregivers’ “COVID-19 Patient Impact”). The high completion rate of the “Every Other Day” survey by caregivers at the onset of the study began to decline starting at the fourth of 14 expected surveys. By the ninth survey, only 76% of the caregivers completed it (Supplement 2).

### Time to complete individual app activities

The average time it took participants to complete the app activities are provided in Table 3. The average time for the cancer patients to complete app activities at baseline ranged from 0.8 min (SD = 0.5) for the “Fast 4” survey to 4.9 min (SD = 1.8) for “COVID-19 Your Input” survey. Except for the caregivers’ time to complete “App Feedback,” the time to complete app activities declined slightly over time. The time of day when app activities were completed is included in Supplement 3.

**Table 3:** Minutes to complete activities once started, by instance.

App activity	Within active study period				
	Baseline	~ 7 days	~ 14 days	~ 21 days	~ 28 days
	N; Mean (SD)	N; Mean (SD)	N; Mean (SD)	N; Mean (SD)	N; Mean (SD)
Caregivers					
About you	50; 3.0 (1.5)	–	–	–	–
App feedback	50; 3.2 (1.8)	–	50; 3.1 (1.4)	–	32; 2.8 (1.5)
COVID-19 your input	50; 4.0 (1.5)	–	–	–	39; 3.5 (1.8)
Your wellness	50; 2.1 (1.9)	50; 1.6 (1.3)	50; 1.6 (1.6)	41; 1.5 (1.2)	26; 1.2 (0.6)
Care snapshot	50; 1.0 (0.6)	–	50; 0.8 (0.4)	–	31; 0.7 (0.6)
Quick check-in	50; 1.5 (1.2)	–	–	–	43; 1.2 (1.2)
Short symptom report	50; 5.8 (2.6)	50; 4.5 (2.6)	49; 4.6 (3.5)	40; 4.3 (4.3)	25; 4.0 (3.0)
Fast 4	49; 0.9 (0.7)	50; 0.7 (0.7)	50; 0.6 (0.3)	43; 0.5 (0.2)	28; 0.5 (0.3)
COVID-19 patient impact	50; 1.4 (0.6)	–	–	–	41; 1.3 (0.7)
Cancer patients					
About you	50; 2.4 (1.4)	–	–	–	–
App feedback	50; 3.5 (1.5)	–	48; 3.2 (1.3)	–	33; 3.3 (1.4)
COVID-19 your input	48; 4.9 (1.8)	–	–	–	44; 4.4 (1.8)
Quick check-in	50; 2.2 (1.4)	–	–	–	45; 1.8 (0.9)
Short symptom report	50; 4.8 (3.1)	50; 4.5 (5.2)	48; 4.1 (5.9)	45; 3.4 (2.3)	29; 3.7 (2.3)
Fast 4	50; 0.8 (0.5)	50; 0.6 (0.2)	48; 0.6 (0.4)	45; 0.5 (0.3)	26; 0.5 (0.2)
Gait speed	49; 2.4 (0.9)	48; 2.0 (0.6)	46; 1.8 (0.6)	36; 1.9 (0.7)	20; 1.7 (0.3)
Sit and stand	49; 2.8 (3.0)	49; 1.7 (0.7)	45; 1.4 (0.6)	38; 1.5 (1.1)	21; 1.1 (0.3)

*SD* standard deviation.

### Completion of app activities by background characteristics

Although none of the caregivers’ or patients’ characteristics, including age, gender, and race, were significantly associated with completing 80% or more of the app activities during the active study period (Supplement 4), we observed some trends. Adherence was overall high across all age categories among patients and reached 90% in patients ≥ 70 years of age. Notably, while the lowest rate adherence was seen among patients aged 50–69, caregivers in this age group were the most compliant (75%). Higher levels of education (associate degree or higher) were marginally (*p* = 0.0592) associated with lower completion of app activities during the active study period.

Caregiving time commitment also was marginally associated with study adherence at both baseline and end of study. When collapsing the reported caregiving time commitment by caregivers to < 20 h versus ≥ 20 h per week, a different trend emerged; at baseline, the rate of adherence by caregivers who committed < 20 h of caregiving was lower than for those who committed ≥ 20 h (59% vs 73%, respectively). This trend became statistically significant at the end of the study, with 92% adherence among caregivers who committed ≥ 20 h caring for their patients compared with only 56% among caregivers committing < 20 h of care (*p* = 0.034, data not shown).

Adherence among cancer patients with late-stage disease (3 and 4) was higher than adherence among patients diagnosed with early-stage disease (81.6% vs. 58.3%, respectively) (Supplement 4). Inversely, adherence was higher among caregivers of patients with early-stage disease (83.3%) compared to caregivers of patients with late-stage disease (60.5%).

## Discussion

In summary, timestamp metadata analysis showed that both adult cancer patients and their informal caregivers were highly adherent over the one-month study duration, with app activity completion at 86% for cancer patients and 84% for caregivers. The percentage of individual activities completed ranged from 91 to 100% for caregivers, and from 79.6% to 100% for patients. The activities that were the least completed by patients were the active physical assessments, gait speed and the sit to stand activity. We suspect these were difficult for patients to complete when they were not feeling well. Cancer patients often felt fatigued, 94% and 80% at the beginning and end of the study, respectively. The surveys that were least completed by caregivers over time were the “Every Other Day” surveys. We believe these surveys might have been too frequent, leading to caregiver burnout on completing them, or the caregivers saw reporting on their own status as less relevant than that of the cancer patient.

Patients and caregivers showed a comparable overall adherence rate (> 85%) for the weekly PRO-CTCAE and PROMIS Physical Function surveys, suggesting the importance of remote monitoring of patients’ symptoms and side effects to patients and caregivers alike. Adherence increased when we allowed 48 h between when the activity appeared in the app and completion, which suggests that whenever possible, it is important to provide a reasonable reporting time window to allow for activity completion. The average time it took caregivers to complete each app activity ranged from less than a minute to 5.8 min; the average time for patients ranged from less than a minute to 4.9 min. Our results do not support the perception that older patients are less compliant with digital health technologies, as the highest (90%) adherence in cancer patients was among the oldest group (≥ 70 years) of participants. This is consistent with a high level of adherence to using physical trackers found in elderly patients with atrial fibrillation^32^.

Interpreting our findings of results in light of known literature, cancer patient adherence with our app-based approach for treatment remote monitoring falls within the range of other studies of electronic clinical outcomes reporting, including 64.7% average patient adherence for weekly reporting during chemotherapy^33^, 74% median adherence for reporting three times per week during treatment with immune checkpoint inhibitors^7^, and 83% adherence for daily reporting in patients with breast or prostate cancer^34^. The United Kingdom electronic self-reporting of adverse events for patients undergoing cancer treatment eRAPID program found 72.2% patient adherence to adverse-event symptom reporting^13^.

The importance of our results is that this study enhances our knowledge about app engagement by demonstrating that metadata can be utilized to inform clinical teams about engagement behaviors of their study participants related to remote data capture and monitoring. This rapid assessment of study activities does not require accessing and analyzing clinical outcome data. Instead, metadata can be used to generate insights on risks of non-adherence during clinical trial data collection or during standard treatment.

There are several limitations of this study. First, we had a relatively small sample size (n = 50 patient–caregiver dyads), so the study was not powered for testing hypotheses about predictors of and barriers to app usage. In addition, only those patients who responded to the invitation to participate in the study were included, and these patients may have been more likely to be adherent. Second, notifications were sent on set dates and times, irrespective of whether activities were completed. The apps have since been updated to coordinate notifications with when activities are due. Third, this pilot study was not designed to test the clinical workflow related to ePROs collected by the apps. The app report was not integrated into the EHR and responses did not go to healthcare providers for review. Further research is needed to assess the feasibility and effect of transmitting urgent data from the apps to healthcare providers. Fourth, there are two recent studies comparing Kaiser membership with the general population as described in the California Health Interview Survey^35^, and one comparing with the US Census and Behavioral Risk Factor Surveillance System^29^. Both found that compared to the total population in the catchment area, the Kaiser members were similar in race, ethnicity, education and income. However, in this study, we did not attempt to achieve a representative sample of participants. The eligibility criteria for the study skewed the population towards females because breast cancer is frequently treated with IV chemotherapy. We suspect that recruitment via email and the requirement that study participants had to own an iPhone 6 or higher and be English speakers may have skewed the representativeness of participants by race/ethnicity. We have since developed an Android version. Finally, our month-long study was not long enough to demonstrate sustained adherence over longer courses of IV chemotherapy.

This study’s strengths included its demonstration of adherence with mobile health apps in a population undergoing active cancer treatment, with patients receiving IV chemotherapy or immunotherapy—therapies that frequently result in side effects. The sample included patients with a mix of cancer types. Observations were reported by patients through our DigiBioMarC app, and by their caregivers through the TOGETHERCare app. This provides the advantage of a second set of eyes on the patient’s symptoms, potentially providing an additive advantage in accuracy and completeness to improve remote monitoring abilities.

Future directions include incorporating more diverse participants across socio-economic, racial, ethnic, and health and digital literacy levels. This will help us examine whether remote monitoring of this type can reduce persistent health disparities by removing requirements to go to specific study locations, which may not be easily accessible with limited resources or from rural areas. We would also like to compare adherence to mobile apps with emailed or texted ePROs. In addition, based upon multiple responses in the semi-structured interviews, we hypothesize that adherence and engagement will be enhanced if clinicians receive the patient reported outcome data. Some of the team plans to explore how to implement this workflow in a frictionless manner within the EHR, with flags for PROs or activities of concern.

## Conclusions

The high rate of participant adherence (86% for cancer patients and 84% for caregivers) in this timestamp metadata analysis suggests that the DigiBioMarC and TOGETHERCare apps can be used to collect patient- and caregiver-reported outcomes data during intensive treatment.

This study assessed user adherence using app metadata to look at completion of expected activities, time to complete activities, and completion of activities by participant characteristics. We suggest that metadata can be used to generate insights on risks of ePRO and remote monitoring of symptoms, health, and adverse events during clinical trial data collection or during standard treatment.

### Supplementary Information

Supplementary Information.

## Data Availability

This work was conducted, in part, with NIH Small Business Innovation Research (SBIR) funding. Due to the sensitive nature of the data captured, the data release requirements of the KPNC subcontract, and the intellectual property associated with this work, all requests for data should be directed to the Chair of the Publication Committee, Dr. Oakley-Girvan (oakley@stanford.edu or Ingrid@medable.com). Requests for data will be considered by the publication committee under a data use agreement and will follow strict guidelines, including HIPAA regulations, regarding data use and the purpose of the analysis.

## References

[1] Khozin S, Coravos A (2019). decentralized trials in the age of real-world evidence and inclusivity in clinical investigations. Clin. Pharmacol. Ther..

[2] Dorsey ER, Kluger B, Lipset CH (2020). The new normal in clinical trials: Decentralized studies. Ann. Neurol..

[3] Basch E (2022). Effect of electronic symptom monitoring on patient-reported outcomes among patients with metastatic cancer: A randomized clinical trial. JAMA.

[4] Basch E (2017). Overall survival results of a trial assessing patient-reported outcomes for symptom monitoring during routine cancer treatment. JAMA.

[5] Basch E (2016). Symptom monitoring with patient-reported outcomes during routine cancer treatment: A randomized controlled trial. J. Clin. Oncol..

[6] Denis F (2019). Two-year survival comparing web-based symptom monitoring vs routine surveillance following treatment for lung cancer. JAMA.

[7] Msaouel P (2021). Evaluation of technology-enabled monitoring of patient-reported outcomes to detect and treat toxic effects linked to immune checkpoint inhibitors. JAMA Netw. Open.

[8] Trojan A, Huber U, Brauchbar M, Petrausch U (2020). Consilium smartphone app for real-world electronically captured patient-reported outcome monitoring in cancer patients undergoing anti-PD-L1-directed treatment. Case Rep. Oncol..

[9] Min YH (2014). Daily collection of self-reporting sleep disturbance data via a smartphone app in breast cancer patients receiving chemotherapy: A feasibility study. J. Med. Internet Res..

[10] Teckie S (2021). A mobile patient-facing app for tracking patient-reported outcomes in head and neck cancer survivors: Single-Arm Feasibility Study. JMIR Form. Res..

[11] Zini EM (2019). A pilot study of a smartphone-based monitoring intervention on head and neck cancer patients undergoing concurrent chemo-radiotherapy. Int. J. Med. Inform..

[12] Kneuertz PJ (2020). Improving patient engagement, adherence, and satisfaction in lung cancer surgery with implementation of a mobile device platform for patient reported outcomes. J. Thorac. Dis..

[13] Velikova G (2022). Electronic self-reporting of adverse events for patients undergoing cancer treatment: The eRAPID research programme including two RCTs. Progr. Grants Appl. Res..

[14] Aapro M (2020). Digital health for optimal supportive care in oncology: Benefits, limits, and future perspectives. Support. Care Cancer.

[15] Oakley-Girvan I, Yunis R, Longmire M, Ouillon JS (2022). What works best to engage participants in mobile app interventions and e-Health: A scoping review. Telemed. J. E. Health.

[16] Davis SW, Oakley-Girvan I (2017). Achieving value in mobile health applications for cancer survivors. J. Cancer Surviv..

[17] Barello S (2015). eHealth for patient engagement: A systematic review. Front. Psychol..

[18] Pratap A (2020). Indicators of retention in remote digital health studies: A cross-study evaluation of 100,000 participants. NPJ Digit. Med..

[19] Gebert P, Schindel D, Frick J, Schenk L, Grittner U (2021). Characteristics and patient-reported outcomes associated with dropout in severely affected oncological patients: An exploratory study. BMC Med. Res. Methodol..

[20] O'Brien HL, Toms EG (2008). What is user engagement? A conceptual framework for defining user engagement with technology. J. Am. Soc. Inf. Sci. Technol..

[21] Kelders SM, van Zyl LE, Ludden GDS (2020). The concept and components of engagement in different domains applied to eHealth: A systematic scoping review. Front. Psychol..

[22] Madujibeya I, Lennie T, Aroh A, Chung ML, Moser D (2022). Measures of engagement with mhealth interventions in patients with heart failure: Scoping review. JMIR Mhealth Uhealth.

[23] Pham Q (2019). A library of analytic indicators to evaluate effective engagement with consumer mhealth apps for chronic conditions: Scoping review. JMIR Mhealth Uhealth.

[24] Oakley-Girvan, I. *et al.* Usability evaluation of mobile phone technologies for capturing cancer patient-reported outcomes and physical functions. *Digit. Health* **9**, 10.1177/20552076231186515 (2023). 10.1177/20552076231186515 PMC10338665 37456127

[25] Oakley-Girvan I (2023). A novel smartphone application for the informal caregivers of cancer patients: Usability study. PLOS Digit. Health.

[26] Lattard C (2023). Clinical and economic impact of clinical oncology pharmacy in cancer patients receiving injectable anticancer treatments: A systematic review. J. Cancer Res. Clin. Oncol..

[27] Öztürk ES, Kutlutürkan S (2021). The effect of the mobile application-based symptom monitoring process on the symptom control and quality of life in breast cancer patients. Semin. Oncol. Nurs..

[28] Suchodolska G, Senkus E (2022). Mobile applications for early breast cancer chemotherapy-related symptoms reporting and management: A scoping review. Cancer Treat. Rev..

[29] Davis AC, Voelkel JL, Remmers CL, Adams JL, McGlynn EA (2023). Comparing Kaiser permanente members to the general population: Implications for generalizability of research. Perm J..

[30] Apple Research Kit. Active Tasks. http://researchkit.org/docs/docs/ActiveTasks/ActiveTasks.html (2018).

[31] Centers for Disease Control and Prevention. Stopping elderly accidents, deaths and injuries (STEADI). https://www.cdc.gov/steadi/ (2021).

[32] Alharbi M, Straiton N, Smith S, Neubeck L, Gallagher R (2019). Data management and wearables in older adults: A systematic review. Maturitas.

[33] Absolom K (2021). Phase III Randomized controlled trial of eRAPID: eHealth intervention during chemotherapy. J. Clin. Oncol..

[34] Crafoord MT, Fjell M, Sundberg K, Nilsson M, Langius-Eklöf A (2020). Engagement in an interactive app for symptom self-management during treatment in patients with breast or prostate cancer: Mixed methods study. J. Med. Internet Res..

[35] Gordon NP (2020). Similarity of adult Kaiser Permanente members to the adult population in Kaiser Permanente’s Northern California service area: comparisons based on the 2017/2018 cycle of the California Health Interview Survey.

