# Supplementary material for: Mobile app activity engagement by cancer patients and their caregivers informs remote monitoring
Source: Sci Rep. 2024 Feb 9;14:3375. doi: 10.1038/s41598-024-53373-w (PMC10858186; doi:10.1038/s41598-024-53373-w)
Supplement: Supplementary file 1 — Supplementary Information. [file 41598_2024_53373_MOESM1_ESM.docx]

**Supplemental Material:** Mobile app activity engagement by cancer patients and their caregivers informs remote monitoring

**Supplement 1. Percentage of total activities completed during the active study period**

| **Activity Completed** | **Caregivers (n = 50)** | | **Cancer Patients (n = 50)** | |
| --- | --- | --- | --- | --- |
|  | **n** | **Percent** | **n** | **Percent** |
| Less than 50% | 1 | 2.0 | 2 | 4.0 |
| 50 to 59% | 6 | 12.0 | 1 | 2.0 |
| 60 to 69% | 4 | 8.0 | 4 | 8.0 |
| 70 to 79% | 6 | 12.0 | 5 | 10.0 |
| 80 to 89% | 7 | 14.0 | 13 | 26.0 |
| 90 to 100% | 26 | 52.0 | 25 | 50.0 |

**Supplement 2. Caregiver completion of TOGETHERCare “Every Other Day” activities over time**


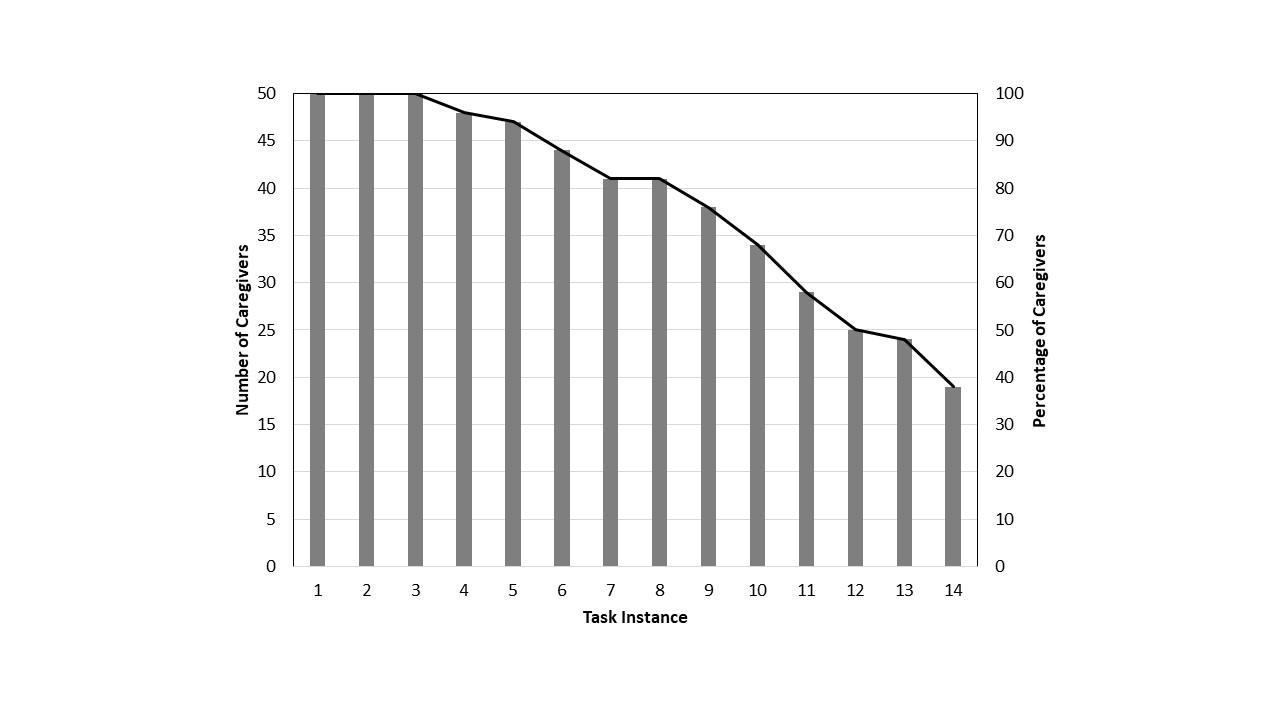


**Supplement 3. Time of day participants interacted with apps during the active study period**

Caregivers and patients interacted with the apps during similar times of day.

**
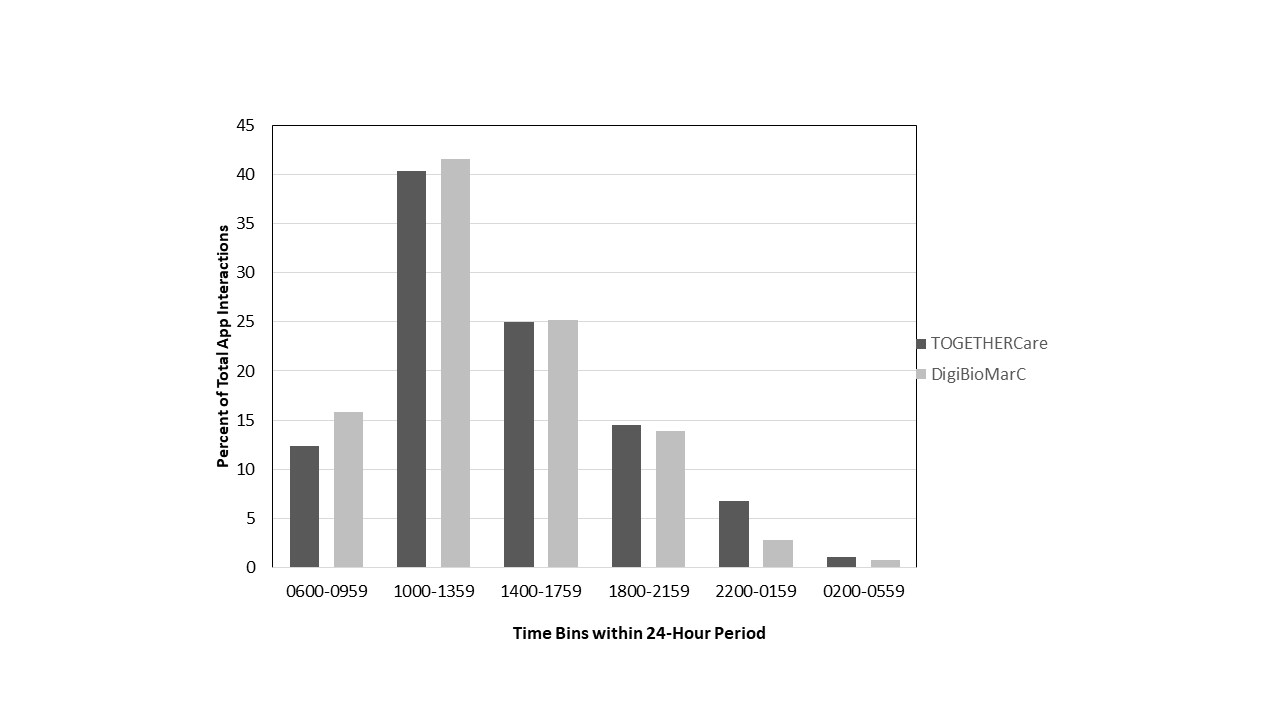
**

**Supplement 4. Participants’ background characteristics and bivariate tests of association between participants characteristics and study adherence defined as whether they completed 80+% of app activities (No/Yes), using Fisher's exact tests**

| **Background Characteristics** | | | **Study Adherence** | | | | | |
| --- | --- | --- | --- | --- | --- | --- | --- | --- |
| **Measure** | **Caregivers (N=50)** | **Patients**  **(N=50)** | **Caregivers** | | | **Patients** | | |
|  | **n (%)** | **n (%)** | **No  n (%)** | **Yes**  **n (%)** | **P value** | **No**  **n (%)** | **Yes**  **n (%)** | ***P* Value** |
| **Age range** | | | | | | | | |
| 20-49 | 17 (34.0) | 10 (20.0) | 8 (47.1) | 9 (52.9) | 0.3124 | 2 (20.0) | 8 (80.0) | 0.3586 |
| 50-69 | 25 (50.0) | 29 (58.0) | 6 (24.0) | 19 (76.0) |  | 9 (31.0) | 20 (69.0) |  |
| 70+ | 8 (16.0) | 11 (22.0) | 3 (37.5) | 5 (62.5) |  | 1 (9.1) | 10 (90.1) |  |
| **Gender** | | | | | | | | |
| Female | 19 (38.0) | 39 (78.0) | 8 (42.1) | 11 (57.9) | 0.3731 | 8 (20.5) | 31 (79.5) | 0.4240 |
| Male | 31 (62.0) | 11 (22.0) | 9 (29.0) | 22 (71.0) |  | 4 (36.4) | 7 (63.6) |  |
| **Ethnicity** | | | | | | | | |
| Hispanic or Latino | 4 (8.0) | 4 (8.0) | 0 (0) | 4 (100) | 0.3076 | 1 (25.0) | 3 (75.0) | 1.0000 |
| Not Hispanic or Latino | 41 (82.0) | 43 (86.0) | 13 (31.7) | 28 (68.3) |  | 9 (20.9) | 34 (79.1) |  |
| Decline to answer | 5 (10.0) | 3 (6.0) | — | — |  | — | — |  |
| **Race** | | | | | | | | |
| White | 32 (64.0) | 34 (68.0) | 11 (34.4) | 21 (65.6) | 0.3563 | 7 (20.6) | 27 (79.4) | 0.2021 |
| Person of Color | 8 (16.0) | 9 (18.0) | 3 (37.5) | 5 (62.5) |  | 3 (33.3) | 6 (66.7) |  |
| Multiracial/ Other | 7 (14.0) | 6 (12.0) | 2 (28.6) | 5 (71.4) |  | 2 (33.3) | 4 (66.7) |  |
| Decline to answer | 3 (6.0) | 1 (2.0) | — | — |  | — | — |  |
| **Education** | | | | | | | | |
| High school/GED | 5 (10.0) | 4 (8.0) | 0 (0) | 5 (100) | 0.3430 | 0 (0) | 4 (100) | 0.0592 |
| Some college | 18 (36.0) | 11 (22.0) | 6 (33.3) | 12 (66.7) |  | 3 (27.3) | 8 (72.7) |  |
| Associate or bachelor’s degree | 17 (34.0) | 20 (40.0) | 6 (35.3) | 11 (64.7) |  | 2 (10.0) | 18 (90.0) |  |
| Graduate degree | 10 (20.0) | 15 (30.0) | 5 (50.0) | 5 (50.0) |  | 7 (46.7) | 8 (53.3) |  |
| **Employment** | | | | | | | | |
| Unemployed | 26 (42.0) | 34 (68.0) | 7 (26.9) | 19 (73.1) | 0.3725 | 9 (26.5) | 25 (73.5) | 0.7278 |
| Employed (full, part, or self) | 24 (48.0) | 16 (32.0) | 10 (41.7) | 14 (58.3) |  | 3 (18.8) | 13 (81.3) |  |
| **Caregiving time per week at baseline** | | | | | | | | |
| <8 hours | 17 (34.0) |  | 5 (29.4) | 12 (70.6) | 0.0696 |  |  |  |
| 9-19 hours | 5 (10.0) |  | 4 (80.0) | 1 (20.0) |  |  |  |  |
| 20-40 hours | 5 (10.0) |  | 1 (20.0) | 4 (80.0) |  |  |  |  |
| >40 hours | 10 (20.0) |  | 3 (30.0) | 7 (70.0) |  |  |  |  |
| Decline to answer | 13 (26.0) |  | 4 (30.8) | 9 (69.2) |  |  |  |  |
| **Caregiving time/week at end of study** | | | | | | | | |
| ≤8 hours/week | 23 (46.0) |  | 8 (34.8) | 15 (65.2) | 0.0768 |  |  |  |
| 9-19 hours/week | 9 (18.0) |  | 6 (66.7) | 3 (33.3) |  |  |  |  |
| 20-40 hours/week | 4 (8.0) |  | 0 (0.0) | 4 (100.0) |  |  |  |  |
| >40 hours/week | 9 (18.0) |  | 1 (11.1) | 8 (88.9) |  |  |  |  |
| Decline to answer | 5 (10.0) |  | 2 (40.0) | 3 (60.0) |  |  |  |  |
| **Patient cancer stage at diagnosis** | | | | | | | | |
| Early (I/II) | — | 12 (24.0) | 2 (16.7) | 10 (83.3) | 0.1811 | 5 (41.7) | 7 (58.3) | 0.1292 |
| Late (III/IV) | — | 38 (76.0) | 15 (39.5) | 23 (60.5) |  | 7 (18.4) | 31 (81.6) |  |

— Not included in bivariate analysis
